# Prognostically relevant natural killer cell-mediated immunoediting in acute myeloid leukemia

**DOI:** 10.1186/2051-1426-1-S1-P181

**Published:** 2013-11-07

**Authors:** Christopher J Chan, Daniel M Andrews, Mark J Smyth, Paul J Neeson, David S Ritchie

**Affiliations:** 1 Immunology, Monash University, Melbourne, VIC, Australia; 2 Cancer Immunology, Peter MacCallum Cancer Centre, Melbourne, VIC, Australia; 3 Sir Peter MacCallum Department of Oncology, University of Melbourne, Melbourne, VIC, Australia; 4 Medicine, University of Queensland, Herston, QLD, Australia

## 

Natural Killer (NK) cells contribute to the control of cancer through immunosurveillance and may influence phenotypic sculpting of cancer through immunoediting. NK cells may also contribute to the control of hematological malignancies such as acute myeloid leukemia (AML) following allogeneic stem cell transplantation. However, no studies have shown direct clinical evidence that supports immunoediting by NK cells in AML at presentation, or whether activating ligand expression at diagnosis serves as a prognostic indicator of survival. We now show that at diagnosis, expression of NK cell ligands on AML blast populations is heterogeneous. Furthermore, expression of multiple activating ligands is associated with favorable cytogenetics and improved leukemia-free survival. In analyses of paired diagnostic and relapse samples, AML blasts exhibiting lower expression of activating ligands were selectively increased at relapse, indicating that NK cell-mediated blast immunoediting occurred prior to AML escape. Therapeutically, NK cell activating ligands could be upregulated on AML by in vitro treatment with bortezomib that enhanced NK cell-mediated cytotoxicity. Thus, diagnostic analyses of the expression of NK cell activating ligands in AML could be used to design therapeutic approaches for specific patients, and agents that stimulate NK cell function by restoring NK cell ligand expression may be appropriate to eliminate minimal residual disease and reduce risk of relapse.

